# Transcriptional regulatory mechanism of *alcohol dehydrogenase 1*-deficient mutant of rice for cell survival under complete submergence

**DOI:** 10.1186/s12284-016-0124-3

**Published:** 2016-09-29

**Authors:** Bijayalaxmi Mohanty, Hirokazu Takahashi, Benildo G. de los Reyes, Edward Wijaya, Mikio Nakazono, Dong-Yup Lee

**Affiliations:** 1 Department of Chemical and Biomolecular Engineering, National University of Singapore, 4 Engineering Drive 4, Singapore, 117585 Singapore; 2 Graduate School of Bioagricultural Sciences, Nagoya University, Furo-cho, Chikusa, Nagoya, 464-8601 Japan; 3 Department of Plant and Soil Science, Texas Tech University, Box 42122, Lubbock, TX 79409-2122 USA; 4 IFReC, Osaka University, 3-1 Yamada-oka, Suita, Osaka 565-0871 Japan; 5 Bioprocessing Technology Institute, 20 Biopolis Way, Centros, Singapore, 138668 Singapore

**Keywords:** Rice, Coleoptile, Submergence, *Alcohol dehydrogenase 1* (*ADH1*), *Reduced alcohol dehydrogenase activity* (*rad*), Promoters, *Cis*-elements, Transcription factors (TFs)

## Abstract

**Background:**

Rice is the only crop that germinates and elongates the coleoptile under complete submergence. It has been shown that *alcohol dehydrogenase 1 (ADH1)-*deficient mutant of rice with *reduced alcohol dehydrogenase activity* (*rad*) and reduced ATP level, is viable with much reduced coleoptile elongation under such condition. To understand the altered transcriptional regulatory mechanism of this mutant, we aimed to establish possible relationships between gene expression and *cis*-regulatory information content.

**Findings:**

We performed promoter analysis of the publicly available differentially expressed genes in *ADH1* mutant. Our results revealed that a crosstalk between a number of key transcription factors (TFs) and different phytohormones altered transcriptional regulation leading to the survival of the mutant. Amongst the key TFs identified, we suggest potential involvement of MYB, bZIP, ARF and ERF as transcriptional activators and WRKY, ABI4 and MYC as transcriptional repressors of coleoptile elongation to maintain metabolite levels for the cell viability. Out of the repressors, WRKY TF is most likely playing a major role in the alteration of the physiological implications associated with the cell survival.

**Conclusions:**

Overall, our analysis provides a possible transcriptional regulatory mechanism underlying the survival of the *rad* mutant under complete submergence in an energy crisis condition and develops hypotheses for further experimental validation.

**Electronic supplementary material:**

The online version of this article (doi:10.1186/s12284-016-0124-3) contains supplementary material, which is available to authorized users.

## Findings

### Transcriptome configuration of the rice *alcohol dehydrogenase 1* (*ADH1*)-deficient mutant

Rice (*Oryza sativa* L.) has the exceptional ability to germinate and elongate the coleoptile under complete submergence. Germination under such condition mainly depends on carbohydrate metabolism and alcoholic fermentation for ATP synthesis by recycling NAD^+^ (ap Rees et al. 1987; Greenway and Gibbs 2003; Bailey-Serres and Voesenek 2008). Alcoholic fermentation is catalyzed by two cytoplasmic enzymes, pyruvate decarboxylase and alcohol dehydrogenase (ADH) to ferment pyruvate to ethanol (Magneschi and Perata 2009). Rice possesses three *ADH* genes: *ADH1*, *ADH2* and *ADH3* (Xie and Wu 1989 and Terada et al. 2007). However, *ADH1* mutant of rice with *reduced adh activity* (*rad* mutant) and much reduced ADH1 protein, is shown to be involved in the suppression of coleoptile elongation under submergence, whereas *ADH2* mutant does not show any suppression in coleoptile elongation (Terada et al. 2007). Hence, the gene *ADH1* is critical for the regeneration of NAD^+^ to sustain glycolysis during elongation of coleoptile under submergence. Matsumura et al. (1998) and Saika et al. (2006) have reported that there was tremendous reduction in ADH activity in *rad* mutant. This reduced functionality of *ADH1* appears to be linked to impaired ATP production and less recycling of NAD^+^. Moreover, involvement of *ADH1* for sugar metabolism via glycolysis to ethanol fermentation has been recently reported (Takahashi et al. 2014). These evidences clearly indicate that a normal level of *ADH1* expression is crucial for coleoptile elongation under submergence.

In the *rad* mutant, the elongation of the coleoptile is slow due to reduced NAD^+^ regeneration and ATP to maintain protein synthesis, cell wall synthesis and membrane proliferation. However, to maintain the balance of metabolites, the *rad* mutant has perhaps evolved control mechanism which may slow down the synthesis of cell building blocks (Hsiao, 1973). Therefore, to elucidate the regulatory mechanism associated with the altered transcriptome of *rad* mutant of rice during germination under complete submergence, we performed an *ab initio* analysis of *cis*-regulatory information content using publicly available microarray data (Takahashi et al. 2011).

## Distribution of putative *cis*-elements among the genes differentially expressed between *rad* mutant and WT rice

To achieve an overall view on the distribution of putative *cis*-elements in the promoters of the differentially expressed genes in *rad* mutant, the motif enrichment analysis was conducted (Additional file 1). The most highly enriched putative *cis*-elements associated with various TFs in the up/or downregulated genes and their total enrichment scores for each TF class are listed in Tables 1, 2 and 3.

**Table 1:** Potential *cis*-elements identified in the promoters of upregulated genes in *rad* mutant

*Cis*-elements	Motifs	Associated TFs	% (TIC), E-value^*^
AT-hook/PE1-like	GAAAAAAAAA	MYB (PF1)	71 (15.87), 9e-004
	TATTTTTTA	MYB (PF1)	58 (14.35), 8e-004
	TTTGTTTTT	MYB (PF1)	52 (13.58), 6e-004
	AAAAAAATG	MYB (PF1)	51 (13.77), 6e-004
GT-element-like	GAAAAAAAAA	MYB (GT-1/GT-3b)	71 (15.87), 9e-004
	GTGTGTTT	MYB (GT-1)	54 (12.50), 7e-004
GARE-like	TTTGTTTTT	MYB (R1, R2R3)	52 (13.58), 6e-004
	TTTACAAA	MYB (R1, R2R3)	56 (12.25), 3e-004
MYB-box-like	AGGTGCACA	MYB (R1, R2R3)	63 (11.06), 5e-004
	TCTCCCAC	MYB (R1, R2R3)	59 (11.66), 3e-003
ABRE-like	AGGTGCACA	bZIP (Gr. A)	63 (11.06), 5e-004
	TCCTCGCC	bZIP (Gr. A)	59 (12.89), 5e-004
As-1-like	AGCATCAA	bZIP (Gr. D, I, S)	71 (10.87), 3e-004
AuxRe-like	TCTCCCAC	ARF	59 (11.66), 3e-003
	TTTGTTTTT	ARF	52 (13.58), 6e-004
MYC-box-like	CCTACTCC	MYC (bHLH)	56 (11.01), 2e-004
	CACATCTC	MYC (bHLH)	54 (11.14), 6e-004
GCC-box-like	CGCCGCCGG	ERF (I, IV, VII, X)	53 (14.54), 4e-004
	GCGGCGGC	ERF (I, IV, VII, X)	51 (14.24), 1e-004
W-box-like	GTGACCAAA	WRKY	61 (10.68) 8e-004
E2F binding site-like	CCCCCGCC	E2F	59 (12.44), 3e-004
PCF1 and PCF2 binding site-like	TCTCCCAC	PCF1 and PCF2 (bHLH)	59 (11.66), 3e-003
ABRE-like	CTCCTCCA	ABI4(AP2)	58 (12.05), 6e-004
Alfin1 binding site-like	GTGTGTTT	Alfin 1 (PHD finger protein)	54 (12.50), 7e-004

* % = percent occurrence among all upregulated genes, *TIC* total information content of homology, *E-value* E-value of homology with promoter database entry

**Table 2:** Potential *cis*-elements identified in the promoters of downregulated genes in *rad* mutant

*Cis*-elements	Motifs	Associated TFs	% (TIC), E-value*
AT-hook/PE1-like	ATATTTTTT	MYB (PF1)	72 (14.31), 3e-004
	TCCAAAAA	MYB (PF1)	71 (11.53), 6e-004
	ATTTTTAAA	MYB (PF1)	58 (13.45), 3e-004
	TTTTTTTCTT	MYB (PF1)	54 (14.95), 6e-004
MYB-box-like	TCCAAAAA	MYB (R2R3, MCB1/2)	71 (11.53), 6e-004
	GATTAGTG	MYB (R2R3)	68 (10.71), 6e-004
	CAACCACA	MYB (R2R3)	51 (11.98), 3e-004
	CCACCCAGC	MYB (R2R3)	51 (12.20), 3e-004
	AAAATCCA	MYB (R2R3)	50 (12.89), 2e-004
GT-element-like	GATTAGTG	MYB (GT-1/GT-3b)	68 (10.71), 6e-004
	TTTTTTTCTT	MYB (GT-1)	54 (14.95), 6e-004
GARE-like	AAAATCCA	MYB (R1, R2R3)	50 (12.89), 2e-004
Pyrimidine-box-like	TTTTTTTCTT	MYB (R1, R2R3)	54 (14.95), 6e-004
	TCTTTTTT	MYB (R1, R2R3	54 (13.57), 3e-004
As-1/ocs/TGA-like	TCCGTCAC	bZIP (Gr. D, I, S)	53 (11.81), 2e-004
	GAAGATGA	bZIP (Gr. D, I, S)	54 (12.78), 2e-004
ABRE-like/G-box-like	CAACCACA	bZIP (Gr A)	51 (11.98), 3e-004
AuxRe-like	GATTAGTG	ARF	68 (10.71), 6e-004
Binding site of the HDZIPs-like	GATTAGTG	HDZIPs	68 (10.71), 6e-004
GCC-box-like	CGGCGGCG	ERF (I, IV, VII, X)	60 (14.85), 6e-004
AAAAG-element-like	TCTTTTT	DOF1/4/11/22	54 (13.57), 3e-004
GAGA element-like	TCTCTCCC	GAGA-binding factor	53(12.93), 8e-004
TATA-box-like	TTTATTTT	TBP	66 (13.19), 4e-004
DBP1 binding site-like	ATTATAATA	DBP1	56 (12.79), 2e-004

* % percent occurrence among all upregulated genes, *TIC* total information content of homology, *E-value* E-value of homology with promoter database entry

**Table 3:** List of transcription factor genes with total enrichment scores among the upregulated (UR) and downregulated (DR) target genes in the *rad* mutant

TF Family	Locus_ID (Annotation)^a^	Fold increase^b^	Total enrichment score of target motifs^c^	
			UR	DR
MYB	Os01g0187900 (Similar to Transcription factor MYBS2)	2.80	587	826
ERF	Os04g0547600 (Pathogenesis-related transcriptional factor and ERF domain containing protein)	6.97	104	60
	Os04g0398000 (Pathogenesis-related transcriptional factor and ERF domain containing protein)	3.86		
	Os04g0550200 (Pathogenesis-related transcriptional factor and ERF domain containing protein)	2.81		
	Os01g0797600 (AP2/ERF family protein, ERF-associated EAR-motif-containing repressor, Abiotic stress response, Stress signaling, OsERF3)	2.62		
WRKY	Os05g0571200 (Similar to WRKY transcription factor 19)	14.59	61	--
	Os01g0826400 (WRKY transcription facto 24)	8.82		
	Os01g0584900 (WRKY transcription factor 28-like (WRKY5) (WRKY transcription factor 77)	5.43		
	Os02g0181300 (Similar to WRKY transcription factor)	4.60		
	Os06g0649000 (Similar to WRKY transcription factor 28)	3.84		
	Os03g0758000 (Similar to WRKY transcription protein)	3.83		
	Os04g0605100 (WRKY transcription factor 68)	3.11		
	Os01g0246700 (Similar to WRKY transcription factor 1)	2.58		
bHLH	Os06g0193400 (Similar to Helix-loop-helix protein homolog)	2.57	59	--

^a^Information based on RAP-DB (http://rapdb.dna.affrc.go.jp/); ^b^Based on microarray data of Takahashi et al. (2011); ^c^total target motif enrichment score = sum of the % occurrences of all motif species belonging to the same TF family in the upregulated (UR) and downregulated (DR) groups of genes (refer to Tables 1 and 2)

**Fig. 1:**
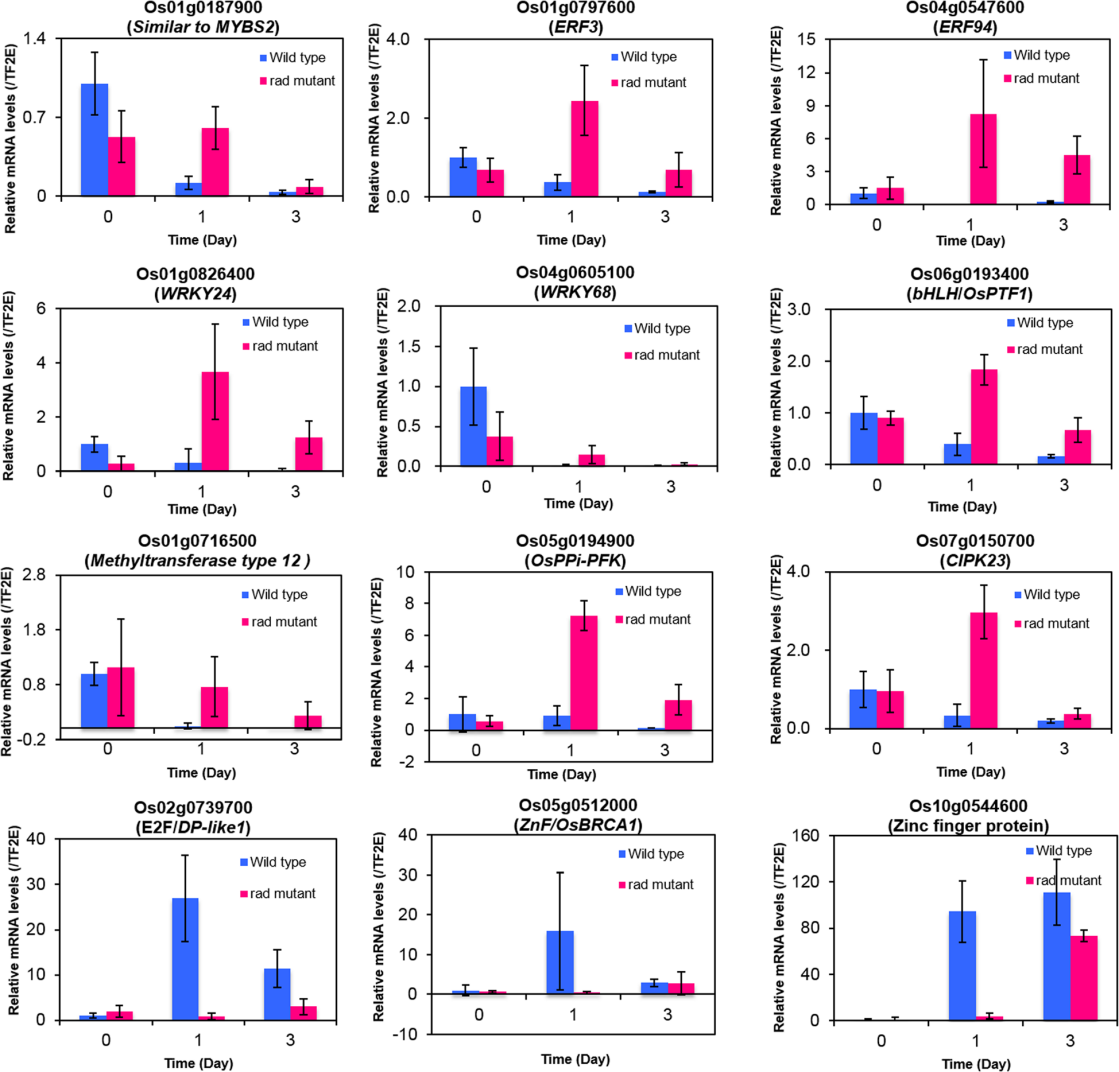
Gene expression pattern of transcription factor, metabolic and signal transduction genes upregulated and downregulated in LM-isolated coleoptiles of *rad* mutant after 0, 1 and 3 days after complete submergence. Rice seeds (*rad* mutant and WT) were germinated under complete submergence for 0, 1 and 3 days after imbibition. Coleoptiles were isolated from rice embryo sections by using LM, and then total RNA was extracted from LM-isolated tissues. Quantitative RT-PCR analysis was performed for selected genes with appropriate primers (Additional file 5: Table S1). Upregulated genes in the *rad* mutant: Os01g0187900 *(Similar to Transcription factor MYBS2),* Os01g0797600 (*ERF3*), Os04g0547600 (*ERF94*), Os01g0826400 (*WRKY 24*), Os04g0605100 (*WRKY 68*), Os06g0193400 *(PTF/bHLH*), Os01g0716500 (*methyltransferase type 12 domain containing protein*), Os05g0194900 (*PPi-PFK*) and Os07g0150700 (*CIPK23*). Downregulated genes: Os02g0739700 (*E2F Family domain containing protein*), Os05g0512000 (*Zinc finger/OsBRCA1*), and Os10g054460 *(*Zinc finger, RING/FYVE/PHD-type domain containing protein). Transcript level of each gene was normalized to the transcript level of rice *TF2E* (Os10g0397200) gene (used as a control gene). Each data point represents the means ± SD (*n* = 3)

In the current analysis, the promoters of the up/downregulated genes in the *rad* mutant were significantly enriched with common putative *cis*-elements which are connected to several TFs such as MYB, bZIP, ERF and ARF. Interestingly, MYB and ERF TF genes are consistently upregulated (Table 3). To confirm their expression level, qRT-PCR analysis was performed for Os01g0187900 (*MYBS2*), Os01g0797600 (*ERF3*), and Os04g0547600 (*ERF94*) at 0, 1 and 3 days after germination under complete submergence for both *rad* mutant and WT (Additional file 1). *MYBS2* and *ERF* showed high expression in *rad* mutant at 1 day and both 1 and 3 days, respectively, after germination (Fig. 1). Hence, these TFs appear to be conserved in both *rad* mutant and wild type and are most likely involved as transcriptional activators (Additional file 2). In contrast, we found significant percentage of putative *cis*-elements associated with WRKY, ABI4, and MYC (bHLH) and high expression level of TF genes (WRKY and bHLH) only in the *rad* mutant (Additional file 2; Table 1). The results suggest that these TFs are possibly involved in the suppression of coleoptile elongation to support cell survival. Among them, qRT-PCR analysis performed for Os01g0826400 (*WRKY 24*), Os04g0605100 (*WRKY 68*) and Os06g0193400 *(bHLH*) showed high expression in *rad* mutant at 1 and 3 days after germination compared to the wild type (Fig. 1). Corresponding with these expression data, we also identified putative w-box-like elements in the promoter regions of some of the key upregulated metabolic genes which might be linked to the reduced coleoptile elongation in *rad* mutant. Examples of such genes are as follows: i) *UDP-glucuronosyl/UDP-glucosyltransferase family protein* (Os06g0220500), which plays a major role in cell homeostasis (Vogt and Jones 2000) and potentially contributes to chemical stability and reduced chemical activity of the cell under stress conditions, ii) *similar to cytochrome P450 family* (Os12g0268000), induced by environmental stresses, which contains binding sites for MYB, MYC, and WRKY in their promoters and is involved in the catabolism of plant hormones (Ayyappan et al. 2015), and iii) *methyltransferase type 12 domain containing protein* (Os01g0716500), which may control the synthesis of cell building blocks by repressing their transcription. Taken together, our results demonstrate a possible link between WRKY TF and metabolic alteration that supports *rad* mutant for the cell survival. Moreover, among the glycolytic genes, *pyrophosphate-dependent phosphofructo-1-kinase-like protein* (*PPi-PFK*) (Os05g0194900) seems to play a major role in energy conservation by using PPi instead of ATP as an alternative energy source during ATP deficiency (Huang et al. 2008). All these genes, having supportive role in the cell survival, also possess common TF binding sites in their promoter regions (Additional file 3: Figure S1). High expression of *methyltransferase type 12 domain containing protein* and *PPi-PFK* performed by qRT-PCR analysis in *rad* mutant at both 1 and 3 days after germination compared to wild type supports their involvement in cell survival (Fig. 1). We also did qRT-PCR analysis for TF genes (downregulated in the mutant) such as Os02g0739700 (*E2F*), Os05g0512000 (*Zinc finger*), and Os10g054460 (Zinc finger protein) at 0, 1 and 3 days after germination under complete submergence for both *rad* mutant and WT (Additional file 1). The expression level of *E2F* (related to cell division) in *rad* mutant was clearly lower than WT at one and three days, and that of *Zinc fingers* (related to protein binding) was much lower at 1 day (Fig. 1). All together, we can hypothesize that the altered transcriptome in the *rad* mutant could be due to a group of candidate transcriptional activators and repressors that may play critical roles under such energy crisis conditions. This is supported by the fact that distinctive TFs act as key activators and repressors in response to various abiotic and biotic stress conditions (Nakashima et al. 2012).

To find the association relationship among genes based on expression data and the candidate TF genes, we developed a gene regulatory network using ARACNE (Margolin et al. 2006). The network clearly provides information regarding the extensive interaction between the regulators and the genes upregulated in the *rad* mutant (Additional file 4: Figure S2).

## Overall analysis of the transcriptome of the *rad* mutant of rice

**Fig. 2:**
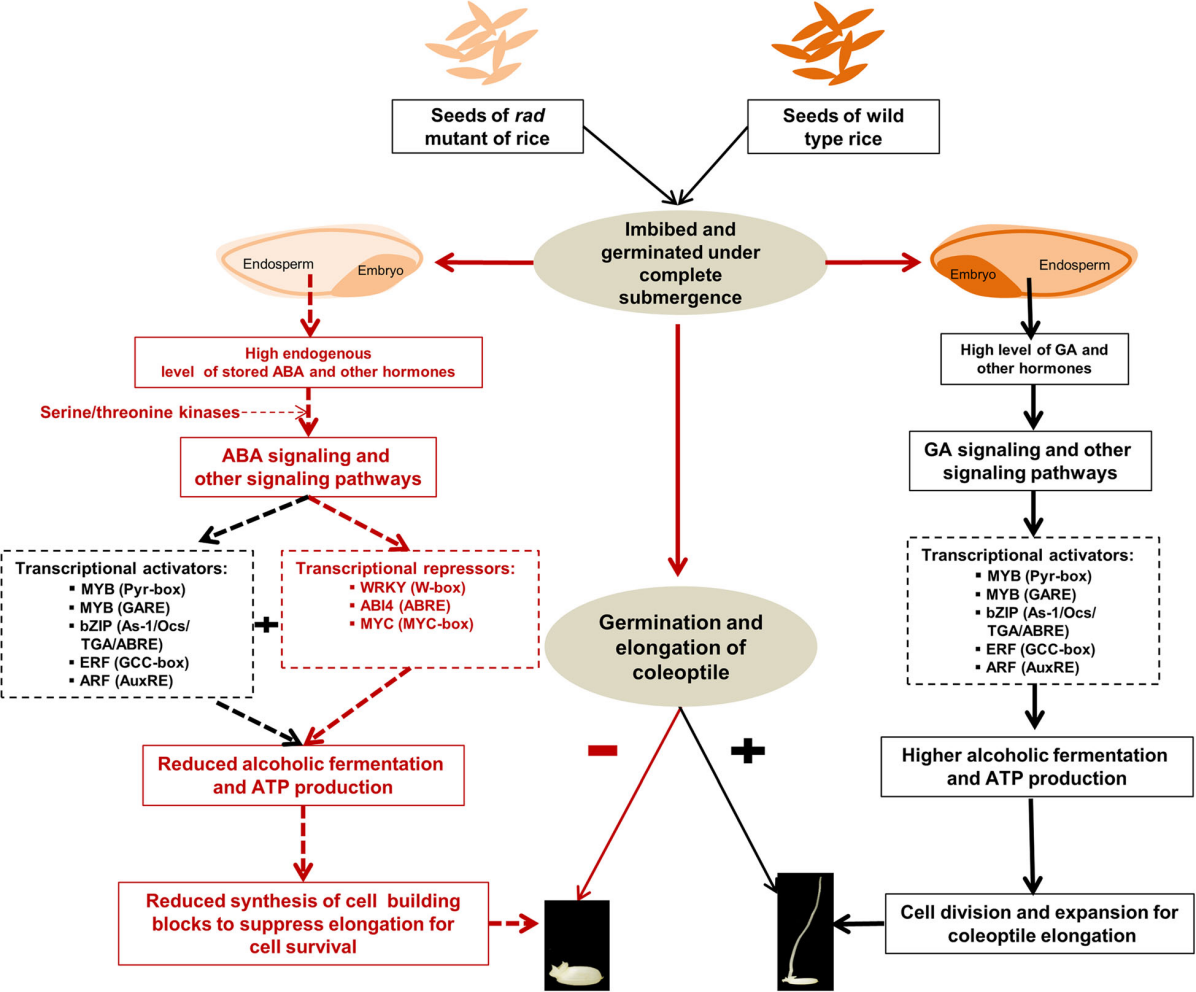
Hypothetical model showing altered transcriptional regulatory mechanisms leading to reduced coleoptile elongation of *rad* mutant. The presence of high endogenous level of ABA in the seeds of *rad* mutant could lead to ABA dependent signaling via serine/threonine kinases. It might lead to the activation of a number of TFs acting as repressors of metabolic genes that slow down the synthesis of cell building blocks for suppression of elongation to maintain metabolites for cell survival. The bigger filled squares with font color in black represent various positive transcriptional modules showing *cis*-elements and their cognate transcriptional regulators. The bigger filled squares with font color in red represent various transcriptional repressors with *cis*-elements and their cognate transcriptional regulators. The dash lines represent the hypothesis predicted from the gene expression data of the *rad* mutant

**Fig. 3:**
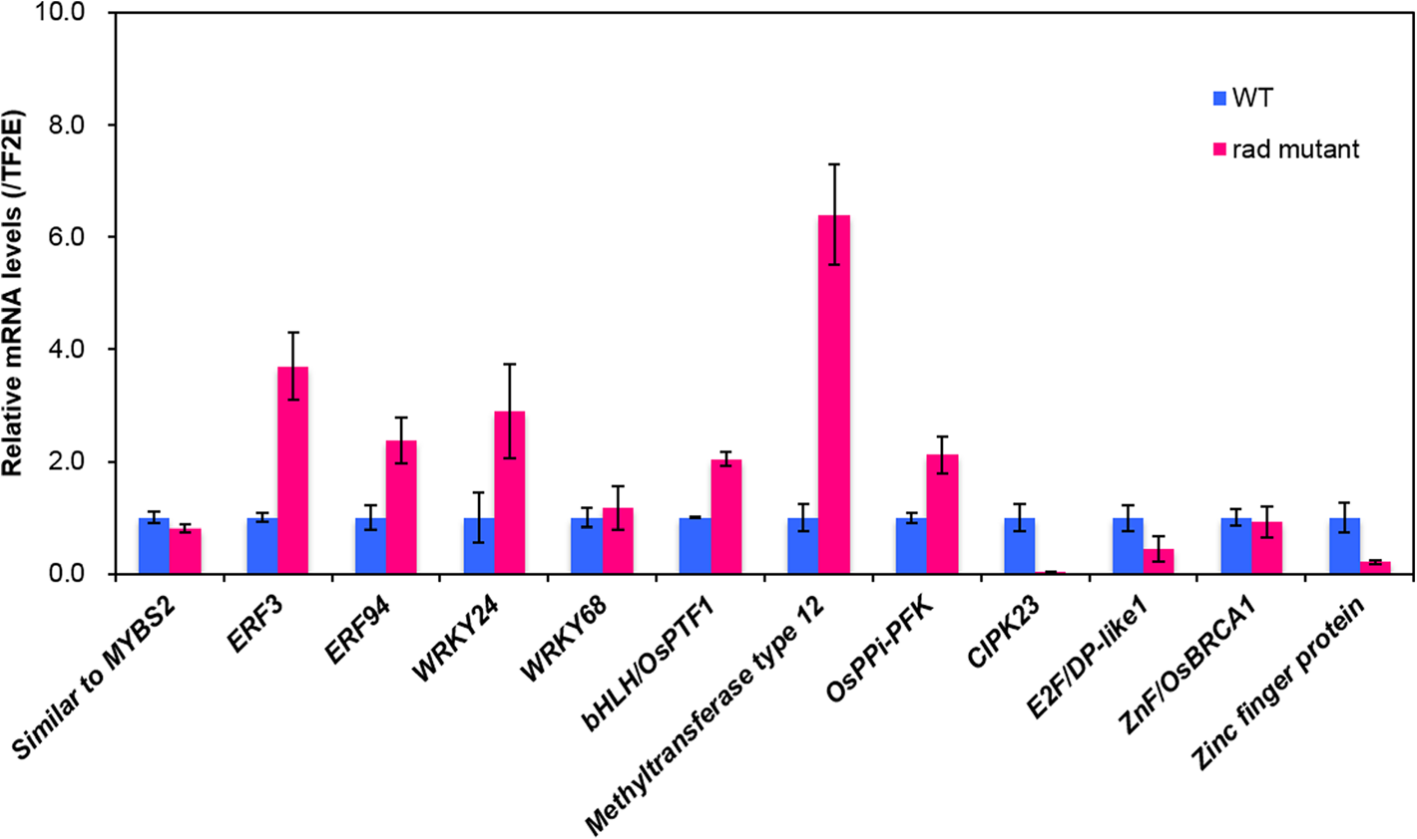
Gene expression pattern of transcription factors, metabolic and signal transduction genes in coleoptiles of *rad* mutant after 7 days under complete submergence. Rice seeds (*rad* mutant and WT) were germinated under complete submergence for seven days after imbibition. Coleoptiles were dissected from rice seedlings, and total RNA was extracted. Quantitative RT-PCR analysis was performed for the selected genes listed in Fig. 1. Transcript levels of each gene were normalized to the transcript levels of rice *TF2E* (Os10g0397200) gene (used as a control gene). Each data point represents the means ± SD (*n* = 3)

Our results evidently demonstrated that reduced *ADH1* activity can affect the regulation of many genes involved in different pathways. A view of how such metabolic changes affected gene expression at the global scale has been established by the patterns of putative *cis*-element enrichment within the affected component of the transcriptome (Haberer et al. 2004). The hypothetical model in Fig. 2 illustrates the possible association of several classes of TFs acting as activators and/or repressors that determine upregulation and downregulation of genes due to the reduced function of *ADH1*. Although putative *cis*-elements associated with MYB TFs were significantly enriched in both *rad* mutant and WT, pyrimidine-box-like elements were absent among the upregulated genes (Tables 1 and 2). Moreover, the *cis*-elements associated with R2R3-MYB-type TFs were more abundant among the downregulated genes, which is similar to the WT where coleoptile elongates under complete submergence. Enrichment of ABRE-like motifs associated with ABI4 TF (Table 1) could be correlated to abscisic acid (ABA) -dependent repression of coleoptile elongation with higher endogenous ABA level as well as interaction of WRKY, ABI4 and MYC (bHLH) TFs as repressor of cell division and elongation. This hypothesis can be linked to the involvement of the upregulation of a number of ABA-dependent abiotic stress responsive serine/threonine kinases in *rad* mutant (Table 4) (Kulik et al. 2011) and higher expression of the *serine/threonine protein kinase* (*CIPK23*) (Os07g0150700) confirmed by qRT-PCR at 1 and 3 days after germination under complete submergence compared to wild type (Fig. 1). The high endogenous level of stored ABA (present in rice seeds) (Mapelli et al. 1995) seems to activate the altered transcriptome for the cell survival. Moreover, to confirm the involvement of genes belonging to different TF families, metabolic and signaling pathways, the qRT-PCR analysis performed for the genes at 0, 1 and 3 days after complete submergence (Fig. 1) was further extended to coleoptiles exposed to complete submergence for 7 days in both *rad* mutant and wild type. The high expression level of most of the genes (upregulated in *rad* mutant) and low expression of the TF genes (upregulated in *rad* mutant) clearly supports their crucial role in cell survival (Fig. 3).

**Table 4:** List of serine/threonine kinase genes upregulated in the *rad* mutant

Locus ID (Annotation)^a^	Description	Function	Fold change (*rad*/WT)
Os07g0150700	Similar to Serine/threonine kinase	Phosphorylation	6.72
Os02g0590800	Similar to Serine/threonine-protein kinase Nek6	Phosphorylation	4.47
Os05g0414700	Serine/threonine protein kinase domain containing protein	Phosphorylation	3.93
Os01g0689900	Serine/threonine protein kinase-related domain containing protein	Phosphorylation	3.47
Os06g0602500	Serine/threonine protein kinase-related domain containing protein	Phosphorylation	3.15
Os10g0431900	Serine/threonine protein kinase-related domain containing protein	Phosphorylation	2.79

^a^Information based on RAP-DB (http://rapdb.dna.affrc.go.jp/)

The bHLH TF family acts as either transcriptional activators or repressors and sometime forms complexes by interacting with MYB and other regulatory proteins that either activate or repress the expression of target genes (Feller et al. 2011). It has been shown that bHLH acts negatively to control seed germination and expansion of cotyledons (Groszmann et al. 2010). In *rad* mutant, it appears to be either directly acting as a repressor or interacting with MYB and other factors to repress the coleoptile elongation. Additionally, identification of w-box associated with WRKY, significant increase in the expression of *WRKY 24* by qRT-PCR analysis, and upregulation of *WRKY 1, 5, 19, 24, 28, 68* and *77* genes (Table 3) altogether support the involvement of WRKY in the reduced coleoptile elongation in the *rad* mutant. Gene regulation occurs mainly due to combinational interaction among different TFs (Istrail and Davidson 2005). Our identification of the *cis*-element distribution and enrichment analysis provides a potentially meaningful view of the combinational role of different TFs in balancing the metabolic status of the *rad* mutant for survival by reducing coleoptile elongation. It also highlights the absence of such regulatory control in the transcriptional network when *ADH1* function is normal.

## Additional files

Additional file 1: Materials and methods. (DOCX 22 kb) Additional file 2: Potential transcriptional activators and repressors that regulate reduced coleoptile elongation under complete submergence. (DOCX 39 kb) Additional file 3: Figure S1. Presence of common putative *cis*-elements in the promoters of key genes in the *rad* mutant. The presence of putative *cis*-elements for binding to potential transcription factors are shown in different strands of the promoter regions (-1000, +200 relative to TSS) of Os06g0220500 (*UDP-glucuronosyl/UDP-glucosyltransferase family protein*), Os12g0268000 (*Similar to cytochrome P450 family*), Os01g0716500 (*Methyltransferase type 12 domain containing protein*), and Os05g0194900 *(pyrophosphate-dependent phosphofructo-1-kinase-like protein)*. TSS: Transcription starts site; T - TATA - box; A - ABI4; B - bZIP (Gr. A); b - bZIP (Gr. D, I, S); E - ERF; H - bHLH; M - MYB and W - WRKY. (TIF 49 kb) Additional file 4: Figure S2. Gene regulatory network of *rad* mutant having reduced *ADH1* activity. A set of key potential TF genes such as, Os01g0826400 *(WRKY transcription facto 24),* Os05g0571200 *(Similar to WRKY transcription factor 19),* Os01g0187900 *(Similar to Transcription factor MYBS2)* and Os04g0547600 *(Pathogenesis-related transcriptional factor and ERF domain containing protein)* involved in the activation and repression of coleoptile elongation showing link to different processes involved in the *rad* mutant. (TIF 1006 kb) Additional file 5: Table S1. Primer list for qRT-PCR. (DOCX 13 kb)

## Abbreviations

ABA: Abscisic acid
*ADH1*: *Alcohol dehydrogenase 1*
*CIPK23*: *Serine/threonine protein kinase*
GA: Gibberellic acid
nt: Nucleotide
*PP*_*i*_*-PFK*: *Ppyrophosphate-dependent phosphofructo-1-kinase-like protein*
*rad*: *Reduced adh activity*
TFs: Transcription factors
TSS: Transcription start site
WT: Wild-type
