# Supplementary material for: Transcriptional regulatory mechanism of alcohol dehydrogenase 1-deficient mutant of rice for cell survival under complete submergence
Source: Rice (N Y). 2016 Sep 29;9:51. doi: 10.1186/s12284-016-0124-3 (PMC5040660; doi:10.1186/s12284-016-0124-3)
Supplement: Additional file 1: — Materials and methods. (DOCX 22 kb) [file 12284_2016_124_MOESM1_ESM.docx]

**Additional file 1**

**Transcriptional regulatory mechanism of *alcohol dehydrogenase 1*-deficient mutant of rice for the cell survival under complete submergence**

Bijayalaxmi Mohanty^1^, Hirokazu Takahashi^2^, Benildo G. de los Reyes^3^, Edward Wijaya^4^, Mikio Nakazono^2^, Dong-Yup Lee^1, 5,*^

**Materials and Methods**

**Identification of genes affected by *alcohol dehydrogenase 1* mutation and extraction of promoter sequences**

Genes that were differentially expressed in the coleoptiles of rice (either upregulated or downregulated) under submerged condition in the *ADH1-*deficient *rad* mutant relative to the wild-type cultivar (Kinmaze) were identified from published microarray data (Takahashi et al. 2011). The promoter sequences [-1000, +200 nt] relative to the experimentally verified TSS for those genes were extracted from our in-house rice promoter sequence database. In total, 261 and 118 promoter sequences were extracted from upregulated and downregulated genes, respectively, which were used for *cis*-element detection.

**Bioinformatics tool for motif detection**

Known and novel promoter motifs were detected by using the Dragon Motif Builder program with EM2 option (Huang et al*.* 2005) according to previously described analysis pipeline (Park et al. 2010; Mohanty et al. 2012; Stamm et al. 2012). Thirty motifs were detected each time having length 8-10 nucleotides per detection at a threshold value of 0.875.

**Biological annotation of putative *cis*-elements**

Motifs with more than 50% occurrence/presence among the subset of candidate promoters were selected at a threshold e-value of ≤10^-3.^ The biological significance of these motifs was verified by their presence in Transcription Factor Binding databases such as TRANSFAC (Matys et al. 2003; [www.gene-regulation.com](http://www.gene-regulation.com/)), PLACE database (Higo et al. 1999; http:www.dna.affrc.go.jp/htdocs/PLACE/) and AGRIS (Davuluri et al. 2003; Yilmaz et al. 2011; <http://arabidopsis.med.ohio-state.edu/>). The percentage occurrences of all motifs belonging to the same TF family were added up to find out the total motif enrichment score according to the method used in Mohanty et al. (2012).

**Plant materials and growth conditions**

The rice (*Oryza sativa*) *rad* mutant and wild type ‘Kinmaze’ were used for the experiments in this study. Dehulled rice seeds were sterilized in 0.6% sodium hypochlorite solution for 30 min. After washing with deionized water 10 times, 10 seeds were placed at the bottom of 1 L glass bottle filled with 1 L deionized water (*i.e.* submerged conditions). Seeds imbibed and germinated under for 0, 1, or 3 days under darkness at 28^0^C. Rice seeds after 0, 1, and 3 days of complete submergence were used for laser microdissection to isolate coleoptile. After 7 days of complete submergence, coleoptiles could be dissected from rice seedling in wild type and *rad* mutant because *rad* mutant slightly elongated coleoptile (Takahashi et al. 2011). Dissected coleoptiles were frozen by liquid nitrogen, and used for the RNA extraction.

**Laser microdissection (LM)**

Rice embryo was dissected from rice seed, and fixed in 75% ethanol: 25% acetic acid. After dehydration in a graded ethanol series, rice embryos were embedded in paraffin and sectioned at a thickness of 10 μm according to Takahashi et al. (2010). Serial sections were placed onto PEN membrane glass slides (Life Technologies, Gaithersburg, MD, USA) for LM as described by Takahashi et al. (2010). To remove paraffin, slides were immersed in 100% Histoclear II (National Diagnostics, Atlanta, GA, USA) for 10 min twice, and followed by air-drying at room temperature. Coleoptile was isolated from rice embryo using a Veritas Laser Microdissection System LCC1704 (Molecular Devices, Sunnyvale, CA, USA).

**RNA extraction**

Total RNA was extracted from the LM-isolated coleoptile using a PicoPure^TM^ RNA isolation kit (Life Technologies) according to the manufacturer’s instructions. The extracted total RNA was quantified with a Quant-iT^TM^ RiboGreen RNA reagent and kit (Life Technologies) according to the manufacturer’s instructions. The quality of total RNA was assessed using a RNA 6000 Pico kit on an Agilent 2100 Bioanalyzer (Agilent Technologies, Santa Clara, CA, USA) as described by Takahashi et al. (2010). Total RNA was extracted from coleoptiles after 7 days of complete submergence using an RNeasy Plant Mini Kit (Qiagen, Hilden,Germany) according to the manufacturer’s instructions.

**Quantitative RT-PCR (qRT-PCR) analysis**

Relative mRNA levels were investigated with qRT-PCR using a StepOnePlus^TM^ real time PCR system (Life Technologies). First-strand cDNA was synthesized using Superscript III (Life Technologies) from 40 ng of total RNA extracted from LM isolated coleoptile. SYBR^®^ Premix Ex Taq™ II (Takara Bio Inc., Shiga, Japan) was used for subsequent PCR amplification with appropriate primers (Additional file 3:Table S1): initial denaturation (95°C for 20 s) and 50 cycles of denaturation (95°C for 5 s), annealing (60°C for 20 s), and extension (72°C for 20 s). Transcript levels of each gene were normalized to the transcript levels of rice *TF2E* (Os10g0397200) gene (used as a control gene).

Relative mRNA levels in coleoptile of 7 days after imbibition were investigated using One Step SYBR^®^ PrimeScript ™ RT-PCR Kit II (Takara Bio Inc.). Five ng total RNA was used for qRT-PCR amplification: Reverse transcription (42°C for 20 minutes) and initial denaturation (95°C for 20 s), 50 cycles of denaturation (95°C for 5 s), annealing (60°C for 20 s), and extension (72°C for 20 s). Transcript levels of each gene were normalized to the transcript levels of rice *TF2E* gene.

**Gene regulatory network analysis**

Gene regulatory network for a set of key transcription factor genes such as, Os01g0826400 *(WRKY transcription facto 24),* Os05g0571200 *(Similar to WRKY transcription factor 19),* Os01g0187900 *(Similar to Transcription factor MYBS2)* and Os04g0547600 *(Pathogenesis-related transcriptional factor and ERF domain containing protein)* involved in the activation and repression of coleoptile elongation in *rad* mutant were constructed using Algorithm for the Reconstruction of Accurate Cellular Networks (ARACNE) (Margolin et al. 2006). ARACNE uses the mutual information of the features to determine the connection between genes. The features included for generating these networks were differential gene expression levels. Based on ARACNE output, the final gene network graphs were created using Cytoscape (Kohl et al. 2011).

**References**

Davuluri RV, Sun H, Palaniswamy SK, Matthews N, Molina C, Kurtz M, Grotewold E (2003) [AGRIS](http://arabidopsis.med.ohio-state.edu/AtcisDB/): Arabidopsis Gene Regulatory Information Server, an information resource of Arabidopsis *cis*-regulatory elements and transcription factors. BMC Bioinformatics 4:25

Higo K, Ugawa Y, Iwamoto M, Korenaga T (1999) Plant *cis*-acting regulatory DNA elements (PLACE) database. Nucleic Acids Res 27:297-300

Huang E, Yang L, Chowdhary R, Kassim A, Bajic VB. 2005. An algorithm for *ab-initio* DNA motif detection. In: Bajic VB, Tan TW, eds. Information processing and living system. World Scientific, Imperial College Press, London, 611–614

Kohl M, Wiese S, Warscheid B (2011) Cytoscape: software for visualization and analysis of biological networks. Methods Mol Biol 696**:**291-303

Margolin AA, Wang K, Lim WK, Kustagi M, Nemenman I, Califano A (2006) Reverse engineering cellular networks. Nat Protoc 1**:**662-671

Matys V, Fricke E, Geffers R, GoBling E, Haubrock M et al. (2003) TRANSFAC: transcriptional regulation, from patterns to profiles. Nucleic Acids Res 31:374-378

Mohanty B, Hearth V, Wijaya E, Reyes BD, Lee DY (2012) Patterns of cis-element enrichment reveal potential regulatory modules in the transcriptional regulation of anoxia response of japonica rice. Gene 511(2):235-242

Park MR., Yun KY, Mohanty B, Herath V, Xu F, Wijaya E, Bajic VB, Yun SJ, de los Reyes BD (2010) Supra-optimal expression of the cold-regulated OsMyb4 transcription factor in transgenic rice changes the complexity of transcriptional network with major effects on stress tolerance and panicle development. Plant cell Environ 33(12):2209-2230

**Stamm P , Ravindran P, Mohanty B, Tan EL, Hao Yu** H**, Kumar PP (2012)** Insights into the molecular mechanism of RGL2-mediated inhibition of seed germination in Arabidopsis thaliana BMC Plant Biol **12**:179

Takahashi H, [Saika](http://aob.oxfordjournals.org/search?author1=Hiroaki+Saika&sortspec=date&submit=Submit) H, Matsumura [H](http://aob.oxfordjournals.org/content/108/2/253.full#aff-3), Nagamura Y, Tsutsumi N, Nishizawa NK, Nakazono M (2011) Cell division and cell elongation in the coleoptile of rice alcohol dehydrogenase 1-deficient mutant are reduced under complete submergence. Ann Bot 108(2):253-261

Takahashi H, Kamakura H, Sato Y, Shiono K, Abiko T, Tsutsumi N, Nagamura Y, Nishizawa NK, Nakazono M (2010) A method for obtaining high quality RNA from paraffin sections of plant tissues by laser microdissection. J of Plant Res123:807–813

Yilmaz A, Mejia-Guerra MK, Kurz K, Liang X, Welch L, Grotewold E (2011) AGRIS: the Arabidopsis Gene Regulatory Information Server, an update. Nucleic Acids Res 39:D1118-D1122
