# Supplementary material for: Transcriptional regulatory mechanism of alcohol dehydrogenase 1-deficient mutant of rice for cell survival under complete submergence
Source: Rice (N Y). 2016 Sep 29;9:51. doi: 10.1186/s12284-016-0124-3 (PMC5040660; doi:10.1186/s12284-016-0124-3)
Supplement: Additional file 2: — Potential transcriptional activators and repressors that regulate reduced coleoptile elongation under complete submergence. (DOCX 39 kb) [file 12284_2016_124_MOESM2_ESM.docx]

**Additional file 2**

**Transcriptional regulatory mechanism of *alcohol dehydrogenase 1*-deficient mutant of rice for the cell survival under complete submergence**

Bijayalaxmi Mohanty^1^, Hirokazu Takahashi^2^, Benildo G. de los Reyes^3^, Edward Wijaya^4^, Mikio Nakazono^2^, Dong-Yup Lee^1, 5,*^

**1. Potential transcriptional activators that regulate coleoptile elongation under complete submergence**

The following transcription factors (TFs) are identified as transcriptional activators that could control the regulation of the germination and elongation of rice coleoptiles under complete submergence.

**MYB Family**

Among the motifs detected, AT-hook/PE1-like, GT-element-like, GARE-like and MYB-box-like associated with MYB TF were highly enriched among the genes that were upregulated in the *rad* mutant relative to the WT. AT-hook/PE1-like elements (MYB, PF1) were the highest scoring among all the MYB -associated motifs. MYB-box (R1, R2R3) and GARE-like elements (R1, R2R3) were equally enriched and were substantially more enriched compared to GT-elements -like motifs (MYB, GT-1/GT-3b). MYB-type TFs are highly conserved in animals, plants and fungi (Lippold et al. 2009) and are known to be involved in the regulation of plant growth and development and different stress responses. The involvement of TF *AtMYB2*, which is known to bind to a MYB-box/GT-element during hypoxic and anoxic responses, has already been confirmed by empirical promoter analysis as well as *in silico* detections (Hoeren et al. 1998; Klok et al. 2002; Dolferus et al. 2003; Liu et al. 2005; Mohanty et al. 2005; Mohanty et al. 2012). The presence of GT motif (5′-TGGTTT-3′) in the promoters of the *aldolase* and ADH1 genes was first identified in maize (Walker et al. 1987). However, the same motif was observed in a reverse orientation i.e. 5’-AAACCA-3’ in the *ADH1* promoter of Arabidopsis (Dolferus et al. 1994). In addition, the role of *ATMYB2* in the regulation of hypoxia in Arabidopsis was also suggested by Liu et al. 2005. The high enrichment and the presence of various MYB-related motifs in our detection suggest the potential involvement of MYB in the germination and growth of rice mutant having reduced *ADH1* activity.

Similarly, motifs such as AT-hook/PE1-like, GT-element-like, GARE-like, pyrimidine-box-like and MYB-box like associated with several classes of MYB TFs were also highly enriched among the genes that were downregulated in the *rad* mutant relative to the wild-type (i.e., upregulated in WT). The enrichment score for MYB-box like elements associated with MYB (R1, R2R3) are much higher among the down regulated genes compared to upregulated genes, implying its potential key role in coleoptile elongation under submergence. This trend is also consistent with our previous results where we identified a high enrichment of MYB-box like elements in the promoters of genes upregulated in rice coleoptiles germinated under anoxia (Mohanty et al. 2012). Reduced coleoptile growth and elongation in the *rad* mutant might have been due to the interaction of some potential TFs that control in suppressing the growth and elongation of coleoptile to maintain the metabolite level for cell survival. In spite of the occurrence of high enrichment of *cis*-elements associated with MYB TFs among the subset of genes that were upregulated in the *rad* mutant, there was reduction of elongation of coleoptile.

**ERF Family**

GCC-box motif associated with Groups I, IV, VII, and X type ERF TFs (Nakano et al. 2006) was moderately enriched in upregulated genes. This enrichment trend could possibly be linked to the expression of a number of ERF-type TFs in the mutant including Os04g0547600 *(Pathogenesis-related transcriptional factor and ERF domain containing protein)*, Os03g0388500 (*Similar to Anther ethylene-upregulated protein ER1, Fragment*) Os04g0398000 (*Pathogenesis-related transcriptional factor and ERF domain containing protein*), Os04g0550200 (*Pathogenesis-related transcriptional factor and ERF domain containing protein*), and Os01g0797600 (*AP2/ERF family protein, ERF-associated EAR-motif-containing repressor, Abiotic stress response, Stress signaling, OsERF3*). The negative role of ERF TF in the regulation of ethylene responsive genes has been demonstrated by a number of studies. It was reported that the japonica cultivar ‘Nipponbare’ that germinates and exhibit normal coleoptile elongation under anoxic conditions does not have such gene and is intolerant to submergence stress (Fukao et al. 2006; Xu et al. 2006). In contrast, the mRNA level of several ERF-like transcription factors was induced in anoxic coleoptiles of japonica rice (Lasanthi-Kudahettige et al. 2007) and seedlings of *Arabidopsis thaliana* (Loreti et al. 2005). In addition, Ismail et al. (2009) established that tolerant genotypes of rice tend to germinate and grow faster with a better seedling survival rates under low oxygen stress, and this was correlated with higher rates of ethylene production. Furthermore, similar *cis*-element analysis of genes upregulated under anoxia in japonica rice (Lasanthi-Kudahettige et al. 2007) revealed that ERF interacts with other hormones in regulating metabolism under anoxia (Mohanty et al. 2012). Although, the major role during anoxic conditions of Arabidopsis ERF genes including *RAP2.2* (Hinz et al. 2010), *HRE1* and *HRE2* (Licausi et al. 2010) and *AtERF73/HRE1* (Yang et al. 2011) has been proposed, the precise role of ethylene during coleoptile elongation in rice is yet to be completely validated.

Likewise, GCC-box motifs associated with Groups I, IV, VII, and X type ERF TFs were also moderately enriched among the genes that were downregulated by complete submergence in the *rad* mutant. Although, this motif class is present in both upregulated and downregulated genes, it still suggests a possible role of ERF TF in the germination and elongation of rice coleoptile under submerged conditions. Furthermore, the coleoptile elongation in the *rad* mutant could be associated with interaction of possible potential TFs acting as repressors.

**E2F Family**

We detected a moderate enrichment of E2F-binding site motifs associated with E2F TFs among the genes that were upregulated in the *rad* mutant. This enrichment trend could be linked to the expression of several E2F-encoding genes including Os08g0502600 (*EF hand domain containing protein*), Os01g0783700 (*Similar to EF-hand Ca2+-binding protein CCD1*), Os09g0482800 (*EF hand domain containing protein*), Os09g0482840 (*EF hand domain containing protein*), Os01g0135700 (*EF-HAND 2 domain containing protein*.), Os06g0683400 (*Similar to EF-hand Ca2+-binding protein CCD1*), Os09g0483100 (*EF-Hand type domain containing protein*), and Os07g0631700 (*EF-Hand type domain containing protein*). The presence of E2F binding sites associated with E2F TF suggests its involvement in the potential regulatory mechanism for cell division essential for coleoptile elongation of rice under anoxia (Kosugi and Ohashi 2002). Even though the *cis*-elements associated with TFs related to cell elongation are detected in *rad* mutant, some other regulatory mechanism might be operational associated with the suppression of coleoptile elongation. Surprisingly, we did not detect any *cis*-elements/motifs associated with E2F TF among the genes that were downregulated in the *rad* mutant although there was high expression of Os02g0739700 (*E2F*).

**bZIP family**

The as-1-like and ABRE-like motifs associated with bZIP TF were moderately enriched in the promoter regions of the genes upregulated in the *rad* mutant. While the expression of any bZIP-type TF was not detected in the dataset, the motif detection results suggest a possible role of ABA in the elongation of coleoptiles. The role of bZIP factor in different abiotic stress signaling has been characterized in few instances including rice. Hossain et al. (2010) showed that *OsABF1* encoding a bZIP TF was induced by abiotic stresses such as anoxia, salinity, drought, cold and ABA in roots and shoots of rice seedlings. This gene is known to bind to the ABA responsive elements (ABRE), suggesting its potential role in ABA-mediated gene expression in rice.

Similarly, we also detected the as-1/TGA-like motifs as well as ABRE-like motifs among the genes that were downregulated in the *rad* mutant. The presence of as-1-like and ABRE like motifs in both the gene subsets that were upregulated or downregulated in the *rad* mutant suggests the possible role of ABA in coleoptile elongation. However, higher level of endogenous ABA in the *rad* mutant might be suppressing the coleoptile elongation.

**ARF family**

The *cis*-element AuxRe associated with the ARF-type TFs was present moderately in both the subsets of genes that were upregulated or downregulated in the *rad* mutant. ARFs are known to be involved mainly in plant growth and development. Although, its role in anoxia/submergence has not been clearly demonstrated yet, there is evidence regarding a cross talk between auxin and abiotic stress response in Sorghum (Wang et al. 2010). The occurrence of putative ARF-associated *cis*-elements in both upregulated and downregulated subsets of genes suggests possible role of ARF-type TFs as a transcriptional regulator of anoxic/hypoxic response transcriptomes in rice.

Additionally, we also identified a moderate enrichment of putative *cis*-elements associated with other TFs such as HDZIP and DOFs among the genes that were downregulated in the *rad* mutant. Involvement of HDZIPs in abiotic stress response mechanism has been demonstrated by Harris et al. (2011), although its precise role in anoxic stress is not understood yet. DOF TF is a class of plant -specific zinc finger domain proteins that bind to a T/AAAAG- core sequence. It has been shown to be involved in the regulation of gene expression during seed germination, gibberellin response in aluerone during post-germination stage, auxin response and other plant defense mechanisms (Riechmann et al. 2000; Yanagisawa 2002; Lijavetzky et al. 2003). The presence of AAAG/AAAAG element in the upregulated genes of WT rice indicates the possible involvement of DOF TF in the regulation of gene expression during seed germination.

Overall, elements associated with MYB, bZIP, ARF and ERF types of TFs are found among genes that were upregulated and downregulated as a result of *ADH1* mutation.

**2. Potential transcriptional repressors that regulate reduced coleoptile elongation under complete submergence**

The following TFs are identified as transcriptional repressors that could control the regulation of the reduced germination and elongation of rice coleoptiles under complete submergence.

**WRKY Family**

The W-box motifs associated with WRKY TFs were identified in 61 % of the promoters of genes that were upregulated in the *rad* mutant. The presence of this motif could be associated with the expression of genes several WRKY-type TFs such as Os05g0571200 (*Similar to WRKY transcription factor 19*),Os01g0826400 (*WRKY transcription facto 24*), Os01g0584900 (*WRKY transcription factor 28-like, WRKY5*, *WRKY transcription factor 77*), Os02g0181300 (*Similar to WRKY transcription factor*), Os06g0649000 (*Similar to WRKY transcription factor 28*) Os03g0758000 (*Similar to WRKY transcription protein*), Os04g0605100 (*WRKY transcription factor 68*), and Os01g0246700 (*Similar to WRKY transcription factor 1*). The W-box motif characterized by the core sequence 5’-TGAC-3’ is essential for the binding to the WRKY TF for its specific role in plant metabolism. It is mostly involved in the positive and negative regulation of biotic stress in defense against pathogens and herbivores via salicylic acid and jasmoic acid signaling pathway (Eulgem 2006; Miao et al. 2007; Peng et al. 2008; Skibbe et al. 2008; Ramamoorthy et al. 2008) as well as abiotic stresses to some extent (Jiang and Deyholos 2006; Zhou et al. 2008; Jiang and Deyholos 2009; Agarwal et al. 2011; Chen et al. 2012). Dong et al. (2003) and Kalde et al. (2003) have reported the role of most of the group III WRKY TFs in response to biotic stresses in Arabidopsis. In rice, induction of WRKY TFs by ABA, NaCl, PEG, cold and heat treatment has already been observed (Xie et al. 2005; Xie et al. 2006; Ramamoorthy et al. 2008; Li et al. 2011; Chen et al. 2012; Lindemose et al. 2013). However, the role of WRKY TFs in anoxia/flooding/submergence stress has not been fully established yet. A class of *OsWRKY* genes such as *OsWRKY72* and *-77* are reported to be involved in the ABA signaling (Xie et al. 2005) and a different class of *OsWRKY51* and *-71* are involved in the suppression of GA induction of the α-amylase gene promoter (Xie et al. 2005; Xie et al. 2006). Besides these roles, a WRKY gene in rice, *OsWRKY24* has been shown to be acting as a novel transcriptional repressor that inhibits both GA and ABA signaling in aleurone cells. Moreover, Ramamoorthy et al. (2008) have reported the role of *OsWRKY24* (LOC_Os01g61080) in the regulation of abiotic stresses in 2-weeks-old seedlings in rice. In our analysis, although there was expression of *OsWRKY 77* that plays a role in ABA signaling, the repressor gene *OsWRKY 24* was also expressed. The reduction of coleoptile elongation in *rad* mutant could possibly be due to the antagonistic effect of GA and ABA mediated by *OsWRKY24*. GA and ABA have been revealed to have antagonistic effect on germination of seeds. This evidence suggests that *OsWRKY24* could be a part of the regulatory network that controls coleoptile elongation in *rad* mutant which needs further experimental verifications. The characteristic non-occurrence of the W-box like motifs among the genes that were downregulated in the *rad* mutant might have some significance with elongation of coleoptiles under complete submergence.

**ABI4 Family**

ABRE-like motifs associated with the ABI4 TF were moderately enriched among the genes that were upregulated in the *rad* mutant. However, we did not find any expression of gene related to ABI4 in the expression dataset examined in this study. ABA is required for the ABI4-dependent induction of *ADH* gene in Arabidopsis (Arenas-Huertero et al. 2000). It is also shown to be involved in transcriptional repressor of sugar-regulated genes. In addition, it has been shown that ABI4 is not required for dormancy but needed for the reduction of germination. Penfield et al. (2006) has observed ABI4 as a repressor of lipid breakdown in the embryo that ultimately represses seed germination by the endosperm. Thus, the repressive role of ABI4 together with the WRKY TFs could be involved in the reduction of coleoptile elongation in the *rad* mutant under submerged condition. The absence of ABRE-like motifs associated with ABI4 among the genes that were downregulated in the *rad* mutant appears to correlate its possible positive effects in the wild-type.

**MYC (bHLH) Family**

A moderate enrichment of MYC-box-like elements associated with MYC -type (bHLH) TFs was established among the genes that were upregulated in the *rad* mutant. TFs belonging to this family have not been studied extensively in plants, but some of them are known to be involved mainly in development and both biotic and abiotic stress responses (Hanano et al. 2008; Fursova et al. 2009; Zhao et al. 2011). MYC TFs are involved in jasmonate-induced defense gene activation in Arabidopsis and tomato (Boter et al. 2004) and ABA signaling and cold stress in Arabidopsis (Abe et al. 2003; Chinnusamy et al. 2003). Recently, it has been observed that genes associated with MYC TFs are also involved in salt and osmotic stress tolerance and controlled by ABA signaling pathway (Ji et al. 2012). Given the link of this TF with ABA signaling, it could be acting as a repressor of coleoptile growth and elongation in the *rad* mutant.

**References**

Abe H, Urao T, Ito T, Seki M, Shinozaki K, Yamaguchi-Shinozaki K (2003) Arabidopsis AtMYC2 (bHLH) and AtMYB2 (MYB) function as transcriptional activators in abscisic acid signaling. Plant Cell 15:63-78

Agarwal P, Reddy MP, Chikara J (2011) WRKY: its structure, evolutionary relationship, DNA-binding selectivity, role in stress tolerance and development of plants. Mol Biol Rep 38:3883–3896

Arenas-Huertero F, Arroyo A, Zhou L, Sheen J, Leon P (2000) Analysis of Arabidopsis glucose insensitive mutants, gin5 and gin6, reveals a central role of the plant hormone ABA in the regulation of plant vegetative development by sugar. Gene Dev 14:2085-2096

Boter M, Ruı´z-Rivero O, Abdeen A, Prat S (2004) Conserved MYC transcription factors play a key role in jasmonate signaling both in tomato and Arabidopsis. GENES & DEVELOPMENT, Cold Spring Harbor Laboratory Press, 18:1577–1591

Chen L, Song Y, Li S, Zhang L, Zou C, Yu D (2012) The role of WRKY transcription factors in plant abiotic stresses. Biochim Biophys Acta 1819:120–128

Chinnusamy V, Ohta M, Kanrar S, Lee B, Hong X, Agarwal M, Zhu JK (2003) A regulator of cold-induced transcriptome and freezing tolerance in Arabidopsis ICE1. Gene Dev 17:1043-1054

Dolferus R, Jacobs M, Peacock WJ, Dennis ES (1994) Differential interactions of the promoter element in the stress response of Arabidopsis *Adh* genes. Plant Physiol 105: 1075-1087

**Dolferus R, Klok EJ, Delessert C, Wilson S, Ismond KP, Good AG,** et al***.* (**2003**)** Enhancing the anaerobic response. Ann Bot 91:111-117

Dong J, Chen C, Chen Z (2003) Expression profiles of the Arabidopsis WRKY gene superfamily during plant defense response. Plant Mol Biol 51:21-37

Eulgem T (2006) Dissecting the WRKY web of plant defense regulators. PLoS Pathog 2(11):e126

Fukao T, Xu KN, Ronald PC, Bailey-Serres J (2006) A variable cluster of ethylene response factor-like genes regulates metabolic and developmental acclimation responses to submergence in rice*.* Plant Cell 18:2021*-*2034

Fursova OV, Pogorelko GV, Tarasov VA (2009) Identification of ICE2, a gene involved in cold acclimation which determines freezing tolerance in Arabidopsis thaliana. Gene 429:98-103

Hanano S, Stracke R, Jakoby M, Merkle T, Domagalska MA, Weisshaar B, Davis SJ (2008) A systematic survey in *Arabidopsis thaliana* of transcription factors that modulate circadian parameters. BMC Genomics 9:e182

Harris JC, Hrmova M, Lopato S, Langridge P (2011) Modulation of plant growth by

HD-Zip class I and II transcription factors in response to environmental stimuli.

New Phytol 190:823-837

Hinz M, Wilson IW, Yang J, Buerstenbinder K, Llewellyn D, Dennis ES, Sauter M, Dolferus R (2010) Arabidopsis RAP2.2: an ethylene response transcription factor that is important for hypoxia survival. Plant Physiol 153(2):757-72

Hoeren FU, Dolferus R, Wu Y, Peacock WJ, Dennis ES (1998) Evidence for a role of AtMYB2 in the induction of the Arabidopsis *alcohol dehydrogenase (ADH1)* gene by low oxygen. Genetics 149:479-490

Hossain MA, [Lee](http://link.springer.com/search?facet-author=%22Yongjoo+Lee%22) Y, [Cho](http://link.springer.com/search?facet-author=%22Jung-Il+Cho%22) JI, [Ahn](http://link.springer.com/search?facet-author=%22Chul-Hyun+Ahn%22) CH, Lee SK, [Jeon](http://link.springer.com/search?facet-author=%22Jong-Seong+Jeon%22) JS, Kang H, [Lee](http://link.springer.com/search?facet-author=%22Choon-Hwan+Lee%22) CH, [An](http://link.springer.com/search?facet-author=%22Gynheung+An%22) G, Park PB (2010) The bZIP transcription factor OsABF1 is an ABA responsive element binding factor that enhances abiotic stress signaling in rice. Plant Mol Biol 72 (4-5):557-566

Ismail AM, Ella ES, Vergara GV and Mackill DJ (2009) Mechanisms associated with tolerance to flooding during germination and early seedling growth in rice (*Oryza sativa*). Ann Bot 103:197-209

Ji L, Wang J, Ye M, Li Y, Guo B, Chen Z, Li H, An X (2012) Identification and Characterization of the *Populus* AREB/ABF Subfamily. J Interg Plant Biol 55(2): 177-186

Jiang Y, Deyholos MK (2006) Comprehensive transcriptional profiling of NaCl-stressed Arabidopsis roots reveals novel classes of responsive genes. BMC Plant Biol 6:25

Jiang Y, Deyholos MK (2009) Functional characterization of Arabidopsis NaCl-inducible WRKY25 and WRKY33 transcription factors in abiotic stresses. Plant Mol Biol 69:91-105

Kalde M, Barth M, Somssich IE, Lippok B (2003) Members of the Arabidopsis WRKY group III transcription factors are part of different plant defence signalling pathways. Mol Plant Microbe In 16:295–305

Klok EJ, Wilson IW, Wilson D, Chapman SC, Ewing RM, Somerville SC et al. (2002) Expression profile analysis of the low-oxygen response in Arabidopsis root cultures*.* Plant Cell 14:2481*-*2494

Kosugi S, Ohashi Y (2002) E2F sites that can interact with E2F proteins cloned from rice are required for meristematic tissue-specific expression of rice and tobacco proliferating cell nuclear antigen promoters. Plant J 29(1):45-59

Lasanthi-Kudahettige R, Magneschi L, Loreti E, Gonzali S, Licausi F, Novi G et al. **(**2007) Transcript profiling of the anoxic rice coleoptile. Plant Physiol 144:218-231

Lertwattanasakul N, et al. (2007) Comparison of the Gene Expression Patterns of Alcohol Dehydrogenase Isozymes in the Thermotolerant Yeast Kluyveromyces marxianus and Their Physiological Functions. Biosci Biotech Bioch 71(5):1170-82

Licausi F*,* van Dongen JT*,* Giuntoli B*,* Novi G*,* Santaniello A*,* Geigenberger P*,* Perata, P (2010) *HRE1* and *HRE2*, two hypoxia-inducible ethylene response factors, affect anaerobic responses in *Arabidopsis thaliana*. Plant J 62:302*-*315

Li S, Fu Q, Chen L, Huang W, Yu D (2011**)** *Arabidopsis thaliana* WRKY25, WRKY26, and WRKY33 coordinate induction of plant thermotolerance. Planta 233:1237-1252

Lijavetzky D, Carbonero P, Vicente-Carbajosa J (2003) Genome-wide comparative phylogenetic analysis of the rice and Arabidopsis Dof gene families. BMC Evol Biol 3(1):17

Lindemose S, O'Shea C, Jensen MK, Skriver K (2013) Structure, function and networks of transcription factors involved in abiotic stress responses. Int J Mol Sci 14(3):5842-78

Lippold F, Sanchez DH, Musialak M, Schlereth A, Scheible WR, Hincha DK, Udvardi MK (2009) *AtMyb41* regulates transcriptional and metabolic responses to osmotic stress in Arabidopsis. Plant Physiol 149:1761-1772

Liu FL, VanToai T, Moy LP, Bock G, Linford LD, Quackenbush J (2005) Global transcription profiling reveals comprehensive insights into hypoxic response in Arabidopsis. Plant Physiol 137:1115-1129

Loreti E, Poggi A, Novi G, Alpi A, Perata P (2005) A genome-wide analysis of the effects of sucrose on gene expression in Arabidopsis seedlings under anoxia. Plant Physiol 137:1130-1138

Miao Y, Zengtgrad U (2007) The antagonist function of Arabidopsis WRKY53 and ESR/ESP in leaf senescence is modulated by the jasmonic acid and salicylic acid equilibrium. Plant Cell 19:819-830

Mohanty B, Krishnan SPT, Swarup S, Bajic VB (2005) Detection and preliminary analysis of motifs in promoters of anaerobically induced genes of different plant species. Ann Bot 96:669-681

Mohanty B, Hearth V, Wijaya E, Reyes BD, Lee DY (2012) Patterns of *cis*-element enrichment reveal potential regulatory modules in the transcriptional regulaton of anoxia response of japonica rice. Gene 511(2):235-242

Nakano T, Suzuki K, Fujimura T, Shinshi H (2006) Genome-wide analysis of the ERF gene family in Arabidopsis and rice. Plant Physiol 140:411-432

Penfield S, Li Y, Gilday AD, Graham S, Graham IA (2006) Arabidopsis ABA INSENSITIVE4 Regulates Lipid Mobilization in the Embryo and Reveals Repression of Seed Germination by the Endosperm. The Plant Cell 18:1887-1899

Peng Y, Batley LE, Chen X, Dardick C, Chem M, Ruan R, Canlas P, Ronald PC (2008) OsWRKY62 is a negative regulator of basal Xa21-mediated defense against *Xanthomonas oryzae pv. oryzae* in rice. Mol Plant-Microbe In18:446-458

Ramamoorthy R, Jiang SY, Kumar N, Venkatesh PN, Ramachandran S (2008) A comprehensive transcriptional profiling of the WRKY gene family in rice under various abiotic and phytohormone treatments. Plant Cell Physiol 49:868-879

Riechmann JL Heard J, Martin G, Reuber L, Jiang C-Z, Keddie J et al. (2000) Arabidopsis Transcription Factors: Genome-Wide Comparative Analysis Among Eukaryotes. Science 290:2105-2110

Skibbe M, Qu N, Galis I, Baldwin IT (2008) Induced plant defenses in the natural environment: Nicotiana attenuata WRKY3 and WRKY6 coordinate responses to herbivory. Plant Cell 20:1984-2000.

Walker JC, Howard EA, Dennis ES, Peacock WJ (1987) DNA sequences required for anaerobic expression of the maize *alcohol dehydrogenase 1* gene. P Natl Acad Sci USA 84:664-668

[Wang](http://link.springer.com/search?facet-author=%22SuiKang+Wang%22) SK, Bai YH, [Shen](http://link.springer.com/search?facet-author=%22ChenJia+Shen%22) CJ, [Wu](http://link.springer.com/search?facet-author=%22YunRong+Wu%22) YR, [Zhang](http://link.springer.com/search?facet-author=%22SaiNa+Zhang%22) SN, [Jiang](http://link.springer.com/search?facet-author=%22DeAn+Jiang%22) DA, [Guilfoyle](http://link.springer.com/search?facet-author=%22Tom+J.+Guilfoyle%22) TJ, [Chen](http://link.springer.com/search?facet-author=%22Ming+Chen%22) M, [Qi](http://link.springer.com/search?facet-author=%22YanHua+Qi%22) YH (2010) Auxin-related gene families in abiotic stress response in Sorghum bicolor.

Xie Z, Zhang ZL, Zou X, Huang J, Ruas P, Thompson D, Shen QJ (2005) Annotations and functional analyses of the rice WRKY gene superfamily reveal positive and negative regulators of abscisic acid signaling in aleurone cells. Plant Physiol 137:176-189

Xie Z, Zhang ZL, Zou X, Yang G, Komatsu S, Shen QJ (2006) Interactions of two abscisic-acid induced WRKY genes in repressing gibberellin signaling in aleurone cells. Plant J 46:231-242

Xu K, Xu X, Fukao T, Canalas P, Maghirang-Rodriguez R, Heuer S, Ismail A, Bailey-Serres J, Ronald PC, Mackill DJ (2006) Sub1A encodes an ethylene responsive-like factor that confers submergence tolerance to rice. Nature 442:705-708

Yanagisawa S (2002) The Dof family of plant transcription factors. Trends Plant Sci 7:555-560

Zhao Y, Zhou LM, Chen YY, Yang SG, Tian WM (2011) MYC genes with differential responses to tapping, mechanical wounding, ethrel and methyl jasmonate in laticifers of rubber tree. J Plant Physiol 168:1649-1658

Zhou QY, Tian AG, Zou HF, Xie ZM, Lei G, Huang J, Wang CM, Wang HW, Zhang JS, Chen SY (2008) Soybean WRKY-type transcription factor genes, GmWRKY13, GmWRKY21, and GmWRKY54, confer differential tolerance to abiotic stresses in transgenic Arabidopsis plants. Plant Biotechnol J 6:486-503

Yang CY*,* Hsu FC*,* Li JP*,* Wang NN*,* Shih MC (2011) The AP2/ERF transcription factor AtERF73/HRE1 modulates ethylene responses during hypoxia in Arabidopsis. Plant Physiol 156:202-212
