# Supplementary figures and images for: Transcriptional regulatory mechanism of alcohol dehydrogenase 1-deficient mutant of rice for cell survival under complete submergence
Source: Rice (N Y). 2016 Sep 29;9:51. doi: 10.1186/s12284-016-0124-3 (PMC5040660; doi:10.1186/s12284-016-0124-3)

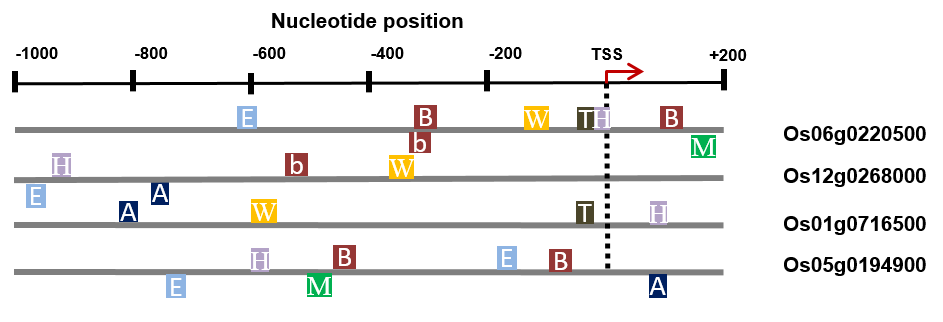

Supplement: Additional file 3: Figure S1. — Presence of common putative cis-elements in the promoters of key genes in the rad mutant. The presence of putative cis-elements for binding to potential transcription factors are shown in different strands of the promoter regions (-1000, +200 relative to TSS) of Os06g0220500 (UDP-glucuronosyl/UDP-glucosyltransferase family protein), Os12g0268000 (Similar to cytochrome P450 family), Os01g0716500 (Methyltransferase type 12 domain containing protein), and Os05g0194900 (pyrophosphate-dependent phosphofructo-1-kinase-like protein). TSS: Transcription starts site; T - TATA - box; A - ABI4; B - bZIP (Gr. A); b - bZIP (Gr. D, I, S); E - ERF; H - bHLH; M - MYB and W - WRKY. (TIF 49 kb) [file 12284_2016_124_MOESM3_ESM.tif]

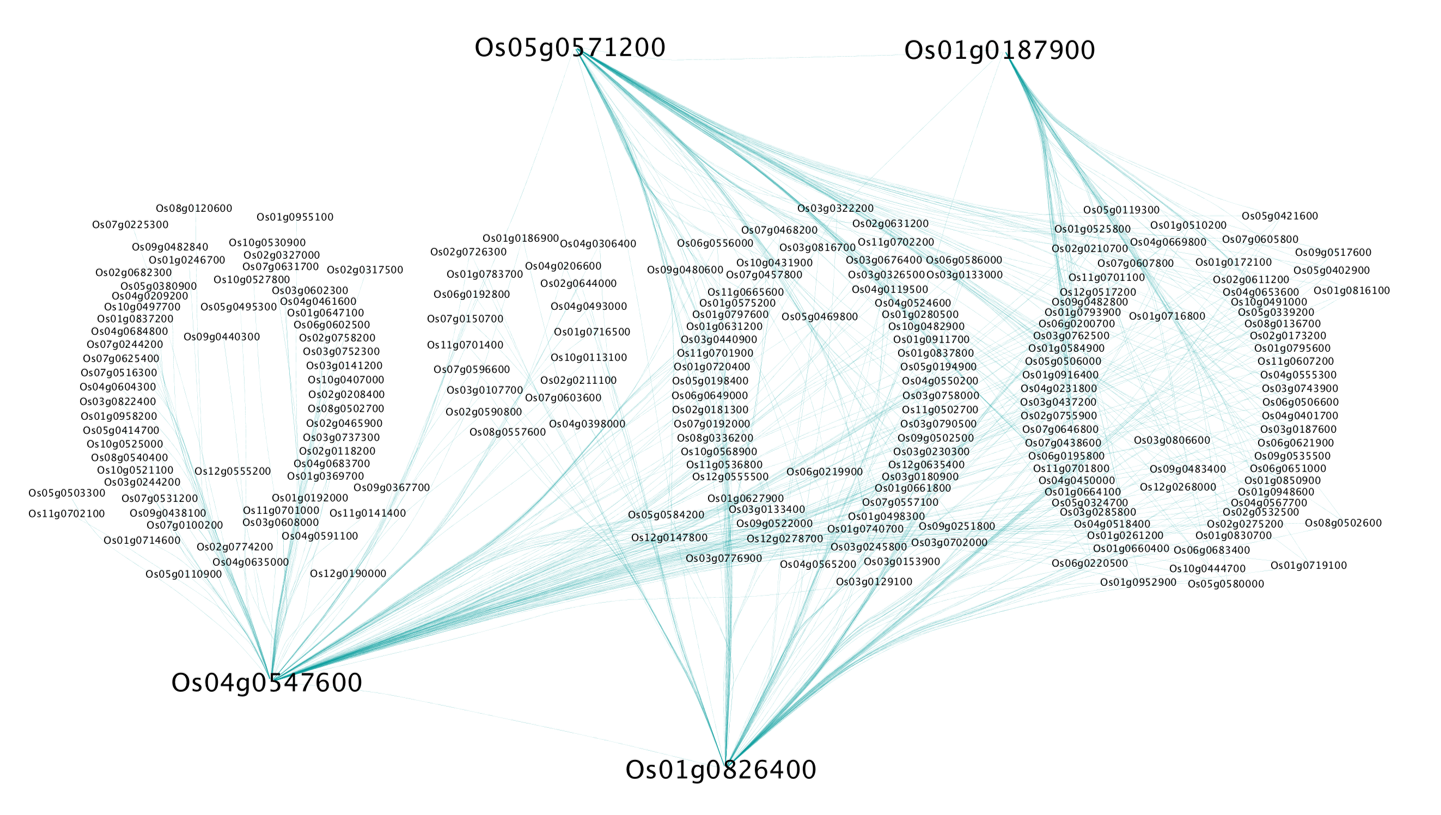

Supplement: Additional file 4: Figure S2. — Gene regulatory network of rad mutant having reduced ADH1 activity. A set of key potential TF genes such as, Os01g0826400 (WRKY transcription facto 24), Os05g0571200 (Similar to WRKY transcription factor 19), Os01g0187900 (Similar to Transcription factor MYBS2) and Os04g0547600 (Pathogenesis-related transcriptional factor and ERF domain containing protein) involved in the activation and repression of coleoptile elongation showing link to different processes involved in the rad mutant. (TIF 1006 kb) [file 12284_2016_124_MOESM4_ESM.tif]
