# Supplementary material for: Transcriptional regulatory mechanism of alcohol dehydrogenase 1-deficient mutant of rice for cell survival under complete submergence
Source: Rice (N Y). 2016 Sep 29;9:51. doi: 10.1186/s12284-016-0124-3 (PMC5040660; doi:10.1186/s12284-016-0124-3)
Supplement: Additional file 5: Table S1. — Primer list for qRT-PCR. (DOCX 13 kb) [file 12284_2016_124_MOESM5_ESM.docx]

**Table S1. Primer list for qRT-PCR**

|  |  |  |
| --- | --- | --- |
| Gene Name | Gene ID | Primer sequence( 5'→3') |
| Similar to MYBS2-fwd | Os01g0187900 | GCCAACATTCTACCCGGCAT |
| Similar to MYBS2-rev | Os01g0187900 | TCCATTCACTGGAGTGGGCT |
| ERF3-fwd | Os01g0797600 | CGATGTTTTGGTCCTCTGTGTTT |
| ERF3-rev | Os01g0797600 | TCCACGGTTTAGTCCATCTCATC |
| ERF94-fwd | Os04g0547600 | TTGGGGTTCCCTCAGCTCAC |
| ERF94-rev | Os04g0547600 | CGCCCCTTCTGTTAAGCTCC |
| WRKY24-fwd | Os01g0826400 | AGCAGCAGAGGCAGAACGA |
| WRKY24-rev | Os01g0826400 | GACGACGATTCTTTCAGTCAGTAGA |
| WRKY68-fwd | Os04g0605100 | GGCAACGGACATGTCTAACCTAA |
| WRKY68-rev | Os04g0605100 | CAAGTTTCCCTCGTCCTCTTTCTAC |
| OsPTF1-fwd | Os06g0193400 | CTTCGGCCATATCCAACCA |
| OsPTF1-rev | Os06g0193400 | GCAGCCGTTGAGTTATTATGTCTTC |
| Methyltransferase type 12-fwd | Os01g0716500 | GCCCAAAGTGCTGAACTGAAA |
| Methyltransferase type 12-rev | Os01g0716500 | CCAATCTATCGCAACGAAACAAG |
| OsPPi-PFK-fwd | Os05g0194900 | CCGCCTGAAGCTTGAAGAA |
| OsPPi-PFK-rev | Os05g0194900 | CATTTCTGTGAATTTCGCACTACC |
| CIPK23-fwd | Os07g0150700 | GCAAATCTAACGGCGACACTC |
| CIPK23-rev | Os07g0150700 | CGGCTTCCACATCACATCTTT |
| DP-LIKE1-fwd | Os02g0739700 | TGGTTTTGTTTATGGGCCTTTC |
| DP-LIKE1-rev | Os02g0739700 | TGGTTTTGTTTATGGGCCTTTC |
| OsBRCA1-fwd | Os05g0512000 | TTCAGAGGAAGCCCGTATCAA |
| OsBRCA1-rev | Os05g0512000 | GCCTTGGATTTTGCCCTATCT |
| Zinc finge proteinr-fwd | Os10g0544600 | GGGAATGGGTGTGAGCAAGT |
| Zinc finger protein-rev | Os10g0544600 | CCTTCCGGTAACTACTCAAGGAC |
| TF2E-fwd | Os10g0397200 | TGCAGCCCAAGGCTAAG |
| TF2E-rev | Os10g0397200 | CGTCGAATAAGCGTAGAGCA |
